# Technical report: Efficient and accurate assessment of neurite outgrowth in spiral ganglion explants using Sholl analysis and repeated measurement ANOVA

**DOI:** 10.1371/journal.pone.0318613

**Published:** 2025-06-04

**Authors:** Christin Geißler, Monika Orsolic, Leon Guchlerner, Marc Diensthuber, Timo Stöver

**Affiliations:** Department of Otolaryngology, University Hospital Frankfurt, Frankfurt am Main, Germany; University of Tartu, ESTONIA

## Abstract

Cultivating three-dimensional spiral ganglion explants is a well-established in-vitro assay for assessing the neurotrophic potential of compounds. The manual neurite measurement remains common but hinders high-throughput experimentation. The present study aimed to automate this process, comparing two methods, Sholl and Gray Value analysis, with manual neurite measurement to enhance this time-consuming and labor-intensive evaluation. The explants were cultured with brain-derived neurotrophic factor (BDNF), and both neurons and neurites were marked immunohistochemically. The comparison of methods included significance of treatment group differences, accuracy, precision, time and interference. Sholl analysis outperformed manual measurements in time and precision, exhibiting fewer interferences compared to Gray Value analysis. It effectively distinguished between control and BDNF concentrations, paralleling manual tracing outcomes. The Sholl intersections per radius analysis, employing repeated measures (rm) ANOVA across 31 measurement points, exhibited the smallest deviation from manual measurement. Gray Value analysis introduced inner explant brightness as a parameter that parallels neuronal survival within the explant. The present study demonstrates, that Sholl analysis with rm ANOVA emerged as the most efficient, with reduced time and manpower requirements. This positions the improved Sholl analysis as a potent tool for high-throughput, automated assessments of neurotrophic potential, marking a significant advancement in the field.

## Introduction

Hearing loss is an escalating issue, affecting 6.1% of the global population [1]. The inner ear can be damaged by factors such as drugs, radiation, or aging [2]. Especially neuronal damage remains irreplaceable. Consequently, the protection and regeneration of the inner ear spiral ganglion neurons have become a crucial area of research [3–7]. Cultivating spiral ganglion explants has emerged as a key method for testing the neurotrophic potential of various compounds on inner ear neurons. To analyze these factors in high-throughput settings, 3D cultures are increasingly being utilized, as they reduce the use of animals while simulating in-vivo conditions.

**Fig 1:**
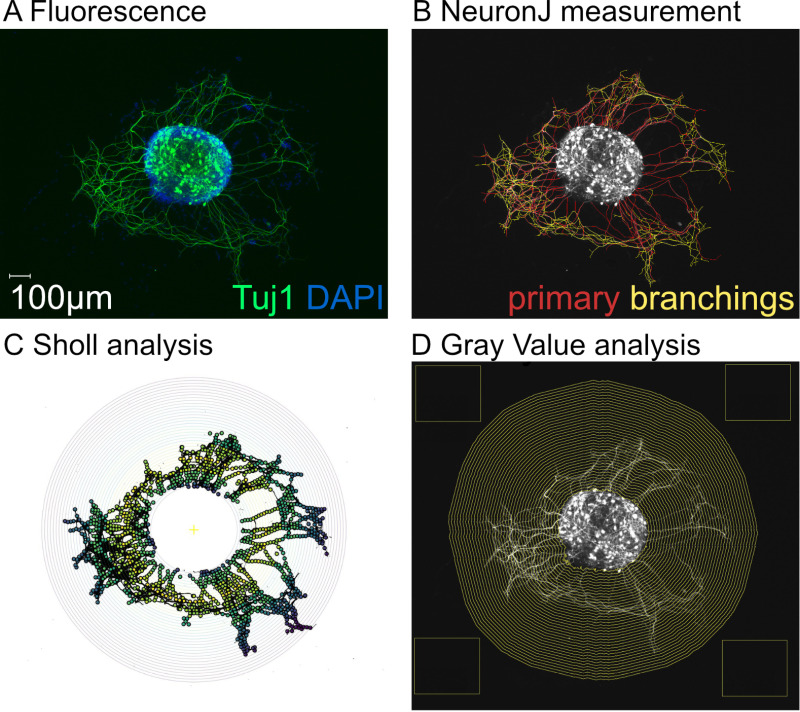
Example image of an explant (experimental run 3, treated with 50 ng/ml BDNF, explant number 4), analyzed using the different methods evaluated in this study. A) Tuj1 staining for neurons and neurites (green), with DAPI nuclear staining (blue). B) Primary neurites (red, the longest branch starting from the explant) and their branches (yellow, branch off from the primary neurites) manually traced using NeuronJ. C) Sholl analysis: A binary image excluded the explant, and circles were drawn around it. These circles, starting at a radius touching all primary neurites, were used to count intersections with neurites, beginning at the explant’s edge. D) Gray Value analysis: Background brightness was assessed using rectangles, and the explant’s outline and brightness were defined. The outline was incrementally expanded to measure the average brightness of each surrounding ring-shaped area.

Within this framework, the quantitative analysis of neurite outgrowth is crucial for determining the effectiveness of neurotrophic substances. However, this evaluation poses significant challenges. Neurites often display irregular and diverse growth patterns from the explant, complicating quantitative analysis. The present study is dedicated to refining the methodology for quantitatively assessing neurite outgrowth. Typically, neurites in spiral ganglion explants are analyzed by evaluating microscopic images (Fig 1).

Manual neurite measurement, though common in examining spiral ganglion explants [3,6,7], is time-intensive and subjective. It involves tracing neurites by hand to record their number and length. Particularly, in high-throughput analyses, this method reaches its limits, requiring considerable time and manpower. Automating this process could save time and increase objectivity.

The aim of this study was to develop an automated measurement to accurately reflect the results of the manual method.

An automated method was developed to measure the fluorescence brightness relative to the distance from explants, assessing the brightness of both explants and neurites (Gray Value analysis). Originating from single neuron studies, Sholl analysis [8] has been adapted in this study for explant neurite growth, counting the intersections of neurites with concentric circles around the explants. The parameter intersections per radius is typically summarized into a single value, such as the sum of intersections or the neurite length index [5]. The neurite length index represents the sum of neurite length measurements and was specifically established by Frick and colleagues for spiral ganglion explants [5]. However, the current study introduces a new method of analyzing Sholl analysis raw data by evaluating the data as an interval of 31 measurement points using repeated measures (rm) ANOVA.

The present study assesses these analysis methods’ effectiveness based on several factors, including the ability to distinguish between treatment groups, the impact of external variables, and the time required for analysis. The impact of the operator on measurements (precision) and the deviation of measurements made with automated methods from manual measurements (accuracy) were also analyzed. In the current study, the well-known brain derived neurotrophic factor (BDNF) [9] was used as a benchmark substance for comparing different evaluation methods. BDNF has demonstrated significant effectiveness in spiral ganglion neurons across numerous studies, making it an important test substance for new factors and methods [10]. In this study, parameters with varying units were standardized as percentages for consistency and normalized against 50 ng/ml BDNF, which reliably produces a significant biological effect.

## Materials and methods

The protocol is available at www.protocols.io under the DOI: dx.doi.org/10.17504/protocols.io.6qpvr853olmk/v1

### Animal preparation

Animal experiments complied with German regulations and EU Directive 2010/63 [§4[3]]. We used Sprague Dawley rats (Janvier, France), with five postnatal day 3–5 rats of both sexes per experiment (n = 3). After decapitation, temporal bones were collected and kept in ice-cold HBSS (Gibco/Thermo Fisher, Germany). Under a stereomicroscope, cochlear capsules were separated, and spiral ganglia were isolated and placed in DPBS (Gibco). These were then cut into explants (~500 µm) and categorized into basal, medial, and apical turns.

### Cell culture

Explants were transferred to chamber slides (eight well, glass bottom, Ibidi, Munich, Germany) coated with laminin (10 µg/ ml; Corning/ Merck, Darmstadt, Germany) and ornithine (0.01% (w/ v), Sigma/ Merck). BDNF (Pepro Tech/ Thermo Fisher) was added to the culture medium (Panserin 401 (PAN Biotech, Aidenbach, Germany), 23.4 mM 4-(2-hydroxyethyl)-1-piperazineethanesulfonic acid buffer (HEPES, Sigma), 0.15% (w/ v) glucose (Sigma), 8.7 µg/ml insulin (Sigma), 0.3x N2 supplement (Life Technologies/ Thermo Fisher), 30 U/ml penicillin (Sigma)) at different concentrations (0 ng/ml = negative control, 10 ng/ml, 25 ng/ml, 50 ng/ml BDNF = positive control). Slides were cultured in an incubator (37 °C, 5% CO_2_) for 72 h and explants were then fixed in acetone-methanol 1:1.

### Immunohistochemistry

The slides were blocked in Dulbecco’s phosphate-buffered saline, pH 7.3 (DPBS, 5% (v/ v) goat serum (Sigma), 1% bovine serum albumin (BSA; Roth, Karlsruhe, Germany), 0.1% (v/ v) Triton X (Sigma)), stained using the neuronal marker beta Tubulin III (anti-Tuj1 antibody AB_2313773. 801202, Bio Legend, San Diego, USA, overnight, 1/2000 in 1xDPBS, 5% goat serum, 1%BSA, 0,1% TritonX) and anti-mouse FITC antibody (goat, 1/200 in DPBS, 0.1% (v/ v) TritonX; AB_2338599, 115-095-146, Jackson Immuno Research, Cambridgeshire, UK). In addition, nucleic 4′,6-diamidino-2-phenylindole (DAPI) staining (1/1000 in DPBS, Invitrogen/ Thermo Fisher) was performed. The fluorescence signal was detected and photographed at constant exposure time with a 5x magnification on an Axio Imager.M2 microscope (Zeiss, Oberkochen, Germany) using AxioVision (Zeiss, version 4.8.1). The explants with their neurites were larger than the microscopic field at 5x magnification. Photos were captured separately, consecutively, with the same exposure time of 8s and without any adjustments. For the measurement of the explants, the microscopic fields of each explant were aligned using Photoshop 2024 (Version 25.11, Adobe Inc., San Jose, California, United States).

### Image analysis

All analyses were performed on a Windows 10 computer with 8 GB of RAM, an onboard graphics card, and a 128 GB hard drive. Analysis was done with ImageJ Fiji (v2.1.0; NeuronJ, Sholl plugins). Excluded were displaced or missing explants. DAPI images identified explant outlines and sizes, whereas immunohistochemical staining against Tuj1 identified neurons and neurites.

For the manual analysis (NeuronJ version 1.4.3 [11], NeuronJ plugin), all primary neurites and their branches were traced. Based on the Tuj1 staining image, the route of the neurites was traced by hand starting from the explant. In the branching of neurites, tracing was performed by following the longest branch (classified as the “primary” branch). Further branches, which branch off from the primary neurites, were identified and traced separately (classified as “branching”). Primary neurites’ number, length, and brightness were measured by four evaluators per explant. Neuronal survival was assessed by counting neurons with visible soma, nucleus, and neurites.

Gray Value analysis quantified fluorescence brightness from the explant outward in 8-bit images. Background brightness, set from four clear edge areas, was subtracted from measured values for the explant itself and the ring-shaped areas around the explant. Explant size was outlined using DAPI staining, expanded by 5 pixels, and applied to Tuji1 images to assess inner explant brightness. The area expanded by 10 pixels (13.21 µm) to 400 µm, calculating mean brightness for each ring-shaped area. Brightness was determined per distance from the explant (= brightness per ring), setting a threshold where brightness per ring dropped below the value one (= threshold distance).

Sholl analysis counts the number of intersections of neurites with circles drawn at regular distances around the explant. Explant outlines, marked and expanded by 5 pixels on DAPI images, were transferred to Tuji1 images for center point calculation and area was cut out. A binary image was formed using a customized brightness threshold above background but below most neurites, with noise reduction applied. The Sholl plugin version 4.1.8 was utilized for analysis [12]. Circles at 10-pixel intervals up to 400 µm were drawn from the center, counting neurite intersections from the first radius covering all primary neurites. The number of intersections per radius, the sum of intersections, mean distance of intersections and neurite length index for each explant was calculated. The difference of intersections between a circle and the next larger circle was calculated and multiplied with the distance of the present circle. The resulting neurite length index described the sum of this neurite length measurements [5].

Gray Value analysis and Sholl analysis were performed using ImageJ Fiji, following the code described in Supplemental 1 file and the raw data are listed in the supplemental raw data Excel file.

### Statistical evaluation

Data analysis was conducted with SPSS (IBM, Version 27) across three independent experiments (n = 3). Values were normalized to a 50 ng/ml BDNF positive control (100%) and reported as original and normalized percentages with SD or 95% confidence interval. Two-tailed tests were used, setting significance at alpha = 0.05.

A one-way ANOVA, corrected by Bonferroni-Holm, evaluated metrics like neurite count, total length, and survival from manual analysis, and Gray Value and Sholl analysis parameters including threshold distance, inner brightness, intersection sum, mean distance, and neurite length index.

Rm ANOVA analyzed distance-dependent Gray Value brightness and Sholl intersections, comparing distances to the explant (within-subject) and BDNF concentrations (between-subject), with Bonferroni-Holm adjusted p-values for multiple comparisons.

Covariates like explant size, cochlear turn, and experimental run were analyzed using one-way ANOVA and Pearson correlation for each method.

To assess inter-rater precision, analysis of Sholl and Gray values for the positive control (50 ng/ml) was performed by three investigators. The intraclass correlation coefficient (ICC) was calculated.

Accuracy was gauged by comparing NeuronJ’s sum of primary lengths (true value) with measurements of the Sholl and Gray Value analysis, presenting mean differences and SDs in a Bland-Altman plot. The calculated data for precision and accuracy are displayed in the supplemental raw data Excel file.

Evaluation duration was assessed with positive control samples (50 ng/ml BDNF). Mean evaluation times per explant, averaged across evaluators, determined overall assessment duration.

## Results

### The Sholl analysis revealed differences between treatment groups comparable to the manual method

**Fig. 2:**
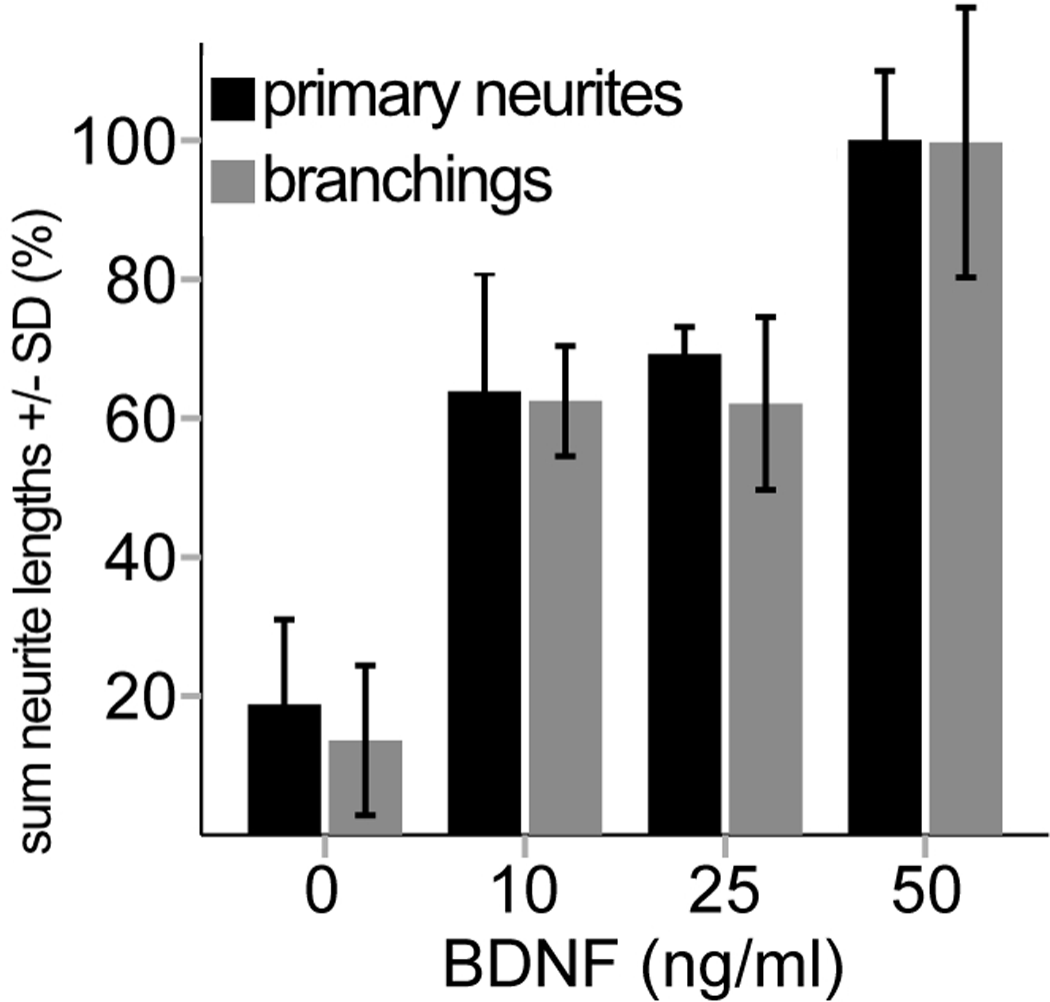
Normalized measurement results from manual neurite tracing with NeuronJ. **Neurite lengths were manually traced and summed separately for primary neurites and their branches. The manual measurement served as the reference method for comparison with the Sholl and Gray Value analyses.** Significant differences were observed between the negative control and the BDNF treatment groups for the sum of primary neurite lengths (10 ng/ml BDNF: p = 0.004; 25 ng/ml and 50 ng/ml BDNF: p < 0.001; ANOVA F(3,8) = 24.23 p < 0.001.

**Fig. 3:**
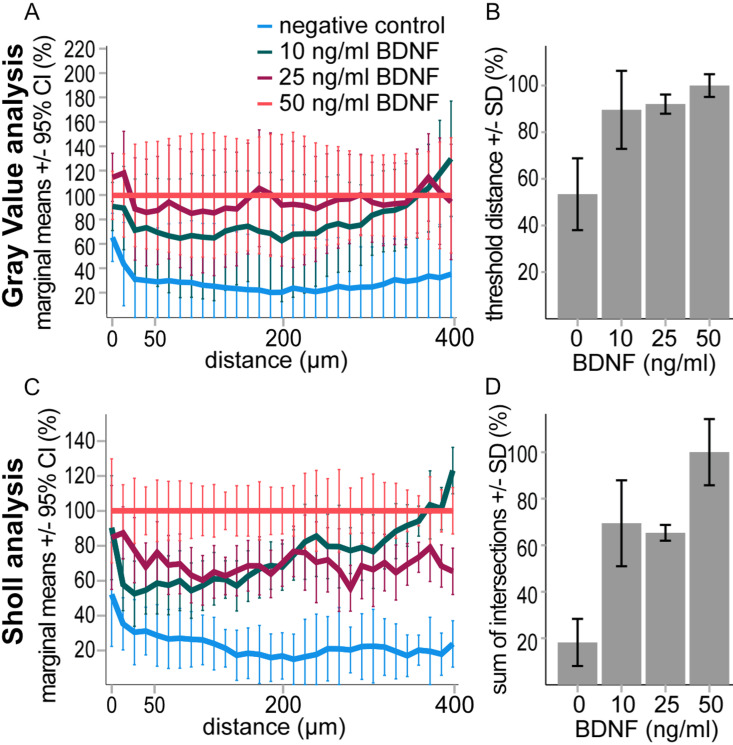
Measurements of Gray Value (A, B) and Sholl analysis (C, D). A) The normalized brightness per ring of the interval ≤ 400 µm is shown (Gray Value analysis). The difference between the negative control and 50 ng/ml BDNF showed a trend (p = 0.06), rm ANOVA F(3,8) = 6.02 p = 0.024. B) Diagram of the threshold distance (Gray Value analysis). There was a significant difference between negative control and 10 ng/ ml BDNF (p = 0.024), 25 ng/ ml BDNF (p = 0.020) and 50 ng/ml (p = 0.006), ANOVA F(3,8) = 9.226 p = 0.006. C) Normalized number of intersections per radius (Sholl analysis), rm ANOVA F(3,8) =23.729 p < 0.001. The difference between negative control and 10 ng/ ml BDNF (p = 0.006), 25 ng/ ml BDNF (p = 0.005) and 50 ng/ml (p < 0.001) was significant. The number of intersections between 50 ng/ml and 10 ng/ ml BDNF (p = 0.05), or 25 ng/ ml BDNF (p = 0.036) was also significant. D) Sum of intersections (Sholl analysis), ANOVA F(3,8) = 20.812 p < 0.001. The difference between negative control and 10 ng/ ml BNDF (p = 0.006), 25 ng/ ml BDNF (p = 0.008) and 50 ng/ ml BNDF (p < 0.001) was significant. The difference between 50 ng/ ml BNDF and 10 ng/ ml BDNF (p = 0.039) or 25 ng/ ml BNDF (p = 0.032) was significant. Additional information in S2 Fig.

In comparison to the negative control, BDNF was found to enhance neurite outgrowth from the explant in a concentration-dependent manner (Fig 2 and 3, S1 Table). The extent of neurite outgrowth was quantified using manual methods, Gray Value analysis, and Sholl analysis. To compare the methods, the outcomes were statistically analyzed to discern differences between the control group and varying concentrations of BDNF. Based on the p values, several parameters for the manual analyzation (sum of primary neurite length, sum of branching lengths, neuronal survival), Gray Value analysis (threshold distance, brightness per ring, inner explant brightness) and Sholl analysis (intersections per radius, sum intersections, neurite length index) were examined for their ability to distinguish the experimental groups.

Within the BDNF treatment groups, significant differences were detected between the 50 ng/ml and 10 ng/ml BDNF (p = 0.009) and between 50 ng/ml and 25 ng/ml BDNF (p = 0.016).

For the sum of branch lengths, significant differences were observed between the negative control and all BDNF concentrations (p < 0.001) and between the 50 ng/ml BDNF group and the lower BDNF concentrations p = 0.003, ANOVA F(3,8)=20.999,p < 0.001. Additional details can be found in S1 Fig.

In the manual analyzation (NeuronJ), the amount and the lengths of the neurites outside the explant (sum of length primary and branchings) were determined (Fig 2, S1 Fig). The most significant difference between the treatment groups was seen in the sum of neurite lengths. A clear distinction could be made between the negative control, low BDNF concentrations (10 ng/ml and 25 ng/ml BDNF) and the highest BDNF concentration. All BDNF treatment groups showed a significant increase in neurite outgrowth compared to the negative control (p ≤ 0.004). The neurite outgrowth was significantly higher at 50 ng/ml BDNF than in the lower BDNF concentrations (p ≤ 0.016). The sum of branching length showed a significant difference between negative control and all BDNF treatments (p < 0.001) and between the low BDNF concentrations and the 50 ng/ml BDNF group (p = 0.003). The number of neurons inside the explant was counted manually. Compared to the negative control, significant more neurons survived in the 25 ng/ml BDNF group (p = 0.014) and 50 ng/ml BDNF (p = 0.037).

In the Gray Value analysis (Fig 3 A,B, S2 Fig), the significant difference of the BDNF groups to the negative control was seen in the parameter threshold distance, that represented the distance at which the brightness falls below the value of one (Fig 3B). All BDNF concentrations showed a significant increase in neurites compared to the negative control (p ≤ 0.024). No significant distinction could be made between the BDNF treatment groups. The results of the brightness per ring showed no significant difference between the treatment groups (Fig 3A). Furthermore, the brightness of the inner explant was measured with Gray Value analysis. The difference between the 25 ng/ml BDNF group and the negative control was significant (p = 0.024). Compared to the manual evaluation of neurite length and neuronal survival, the statistically significance level of the Gray Value analysis was lower.

In Sholl analysis (Fig 3 C,D, S2 Fig), the normalized number of intersections per radius showed the most effective distinction of the treatment groups (Fig 3C). The neurite outgrowth of all BDNF concentrations was significantly higher than the negative control (p ≤ 0.006). The difference between lower and highest BDNF concentrations was also evident from the results (p ≤ 0.05). A similar distinction of treatment groups was given by the sum of intersections (Fig 3D). The difference between negative control and the BDNF groups was significant (p ≤ 0.008) as well as the additional increase in neurite outgrowth between the lower BDNF concentrations and 50 ng/ml BDNF (p ≤ 0.039). The calculation of the neurite length index also showed this significant subdivision of the BDNF groups. The difference between negative control and all BDNF concentrations (p ≤ 0.017) and between the lower and the highest BDNF concentrations (p ≤ 0.021) was evident. All parameters that were calculated from the Sholl analysis had comparatively low p values like the manual analysis and allowed the distinction between the groups: negative control, low BDNF concentrations (10 ng/ml, 25 ng/ml), and 50 ng/ml BDNF.

### The Sholl analysis had fewer interfering factors than the Gray Value analysis

In the Gray Value analysis, the background brightness has a strong influence on the results (test of intermediate subject effect p = 0.011). Staining artifacts were difficult to remove and have a strong influence on the results. The staining intensity between the experiments was significantly different (ANOVA p < 0.001). Additional information is provided in S3 Fig.

In Sholl analysis, the results remain unaffected by staining artifacts or variations in staining intensity between experiments. In the Sholl analysis, a positive correlation was found between the explant size and the intersections per radius (Pearson r = 0.603, p = 0.017). This indicated that the larger an explant was, the more neurites grow out of it.

### Accuracy, precision and analysis time highlighted the Sholl analysis

Accuracy, precision and analysis time were compared between the manual measurement (sum of the primary lengths), Sholl analysis (intersections per radius, sum of intersections, neurite length index) and Gray Value analysis (threshold distance).

**Fig 4:**
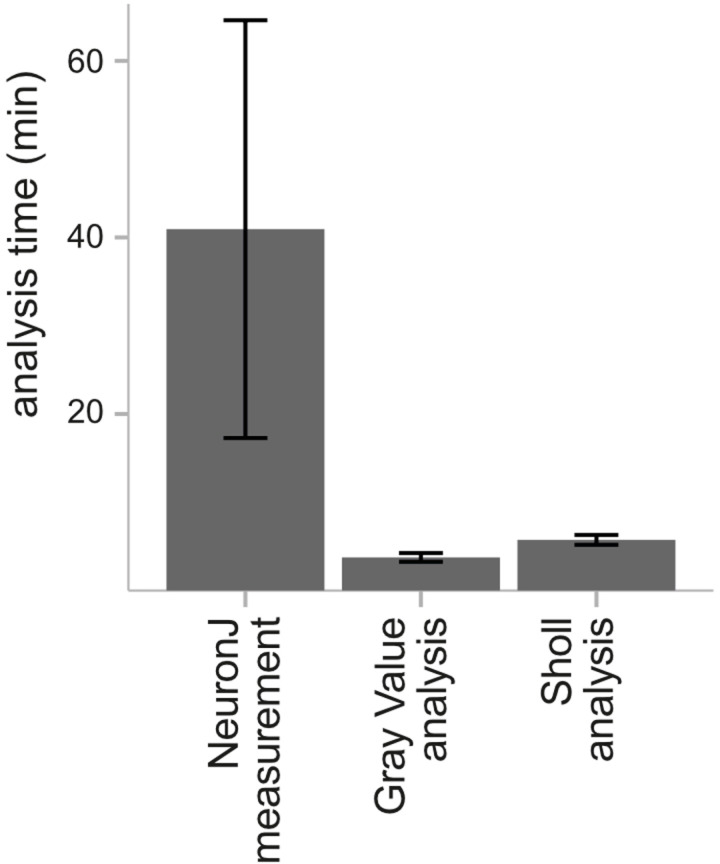
Comparison of analysis time. The differences in analysis times between manual (NeuronJ measurement) and Sholl analysis (p < 0.001), and between manual (NeuronJ measurement) and Gray Value analysis (p < 0.001) were significant.

Analysis time (Fig 4) is an important factor in the laboratory. The manual analysis took 40.9 ± 23.7 min, the Sholl analysis 5.7 ± 0.6 min and the Gray Value analysis 3.8 ± 0.5 min. Sholl and Gray Value analysis were significantly faster than the manual measurement (ANOVA F(2,56) = 45.688 p < 0.001). The variability in manual measurements arises from differing neurite growth between test groups, as measurement time is directly linked to the number of neurites. In contrast, automated measurements require consistent effort.

**Fig 5:**
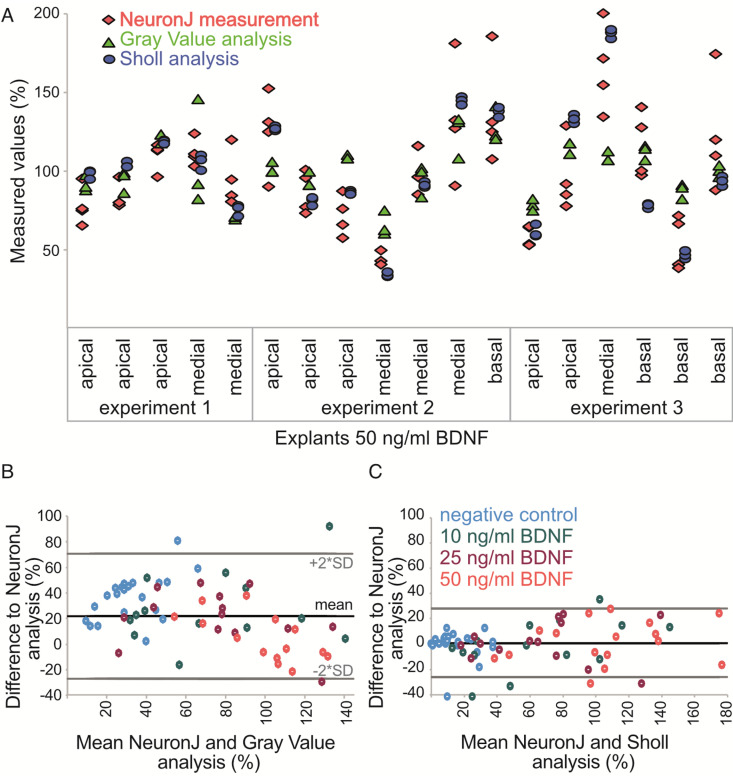
Comparison of precision (A) and accuracy (B,C) of neurite outgrowth. Normalized results of the following parameters were presented: cumulated length of primary neurites (manual measurement), threshold distance (Gray Value analysis), intersections per radius (marginal means, Sholl analysis). Explants were grouped based on cochlear regions, with high frequencies processed at the base, low frequencies at the apex, and intermediate frequencies in the middle region. A) The plot shows the measurements of different investigators for the 50 ng/ml BDNF group. The difference in results between the evaluators was greater for the manual and Gray Value analysis than for the Sholl analysis, where precision was highest. B and C) The Bland-Altman scatter plot represents the difference between measurements of different methods. The manual measurement was used as a true value and compared with Gray Value (B) or Sholl analysis (C). The normalized results for all measured explants of all study groups were shown (negatives in blue, 10 ng/ ml BDNF explants in green, 25 ng/ ml explants in orange, 50 ng/ ml explants in red). Each dot represents one explant. The y-axis shows the difference of the measured values between the methods of analysis. The lines represent the mean (black) and the 2-fold SD (gray). The x-axis shows the mean value between the methods of analysis. The measured values of the Sholl analysis were much closer to the manual measurement than the Gray Value analysis data.

To assess precision (Fig 5A), explants of the positive control (50 ng/ml BDNF) were evaluated by at least 3 evaluators (manual measurement n = 4, Sholl analysis n = 3, Gray Value analysis n = 3). The manual measurement (sum of the primary neurite lengths), Sholl (intersections per radius, sum of intersections, neurite length index) and Gray Value (threshold distance) analysis parameters were compared. The interrater variability between the results of the evaluators was much better (closer to the value of one) for the Sholl intersections per radius and sum of intersections (ICC = 0.995), or neurite length index (ICC = 0.916) than for the Gray Value threshold distance (ICC = 0.723) or manual measurement (ICC = 0.616). The results from the Sholl analysis for the explants had the highest ICC value.

For the assessment of the accuracy (Fig 5B and C), the sum of the primary lengths was set as the true value. The accuracy was presented as the mean difference to the true value with SD. For the Gray value analysis, the measurements of threshold distance deviated by 22 ± 25% from the true value. The results of the Sholl intersections per radius analysis were significantly closer to the true value (1 ± 14%) than those of the Gray Value analysis. The accuracy of the sum of intersections (-6.9 ± 30%) and the neurite length index (-5.3 ± 25%) had a slightly higher deviation than the Sholl intersections per radius. The summary is shown in S2 Table.

## Discussion

Our study advocates for the shift from traditional manual methods to automated analyses like Sholl and Gray Value analysis for neuronal spiral ganglion explants, referencing the manual approaches of Barclay et al. [3], Stolle et al. [6], Bertram et al. [7], and Ma et al. [13]. Sholl analysis, while less common in spiral ganglion research, is validated across various neuronal studies such as those involving the dorsal root ganglion and spinal cord slices [13,14], and retina and optic nerve explants [15], highlighting its standardization and clear data presentation benefits.

The current study found that Sholl analysis yields results comparable to manual methods, suggesting its adoption in spiral ganglion neurite outgrowth assessments due to its time, reliability, and maintained accuracy.

While Sholl analysis is effective, it reduces image detail. Our study explored the automated Gray Value analysis to enhance detail retention, using 8-bit grayscale images for finer resolution than Sholl’s binary method. However, it was less effective in differentiating treatment groups. Sholl analysis showed better consistency and reliability in neurite measurement compared to Gray Value analysis, especially across different investigators.

Sholl analysis matched manual measurements in distinguishing treatment groups but Gray Value analysis was sensitive to interferences like background brightness. Sholl’s binary approach, ignoring background and artifacts, offered more consistency and less susceptibility to such interferences. However, explant size affected Sholl analysis, suggesting outlier exclusion for better accuracy. Despite limitations, precision and reliability of the Sholl analysis make it superior for neurite outgrowth assessments.

Gray Value analysis complements Sholl analysis by measuring inner explant brightness, hinting at neuron survival. Our study found improved survival with BDNF concentrations ≥25 ng/ml. This method offers an objective alternative to manual counting, making it a useful secondary tool alongside Sholl analysis.

The current study employs repeated measures ANOVA (rm ANOVA) to analyze neuronal branching patterns by systematically examining 31 data points within a 400 µm radius around the explant. While traditional Sholl analysis typically condenses complex morphological data into single-value parameters (sum of intersections, neurite length index), this approach preserves the multipoint information structure. By analyzing 31 data points simultaneously, this method provides a more comprehensive and statistically robust comparison, minimizing the information loss associated with data reduction strategies and retaining the full complexity of the original data.

The rm ANOVA enhances statistical reliability by improving test strength, variance estimation, and reducing alpha error accumulation [16], potentially requiring fewer explants and lowering the risk of false negatives, significantly benefiting study results.

The Sholl intersections per radius analysis demonstrated improved accuracy compared to the sum of intersections and neurite length index. Hence, intersections per radius, analyzed with rm ANOVA, emerges as a reliable measure for neurite outgrowth evaluation.

Despite its age, Sholl analysis remains vital, supported by new tools like Spot microscopy software (Diagnostic Instruments) [9], Imaris 8.0 (BitPlane, Switzerland) [17], and Harmony neuron detection algorithm (PerkinElmer) [18] for neurite measurement, which demand specific tech setups. A distinct advantage of using Sholl analysis along with ImageJ Fiji is its adaptability to existing lab technical equipment and its open-source nature, granting researchers full insight and the capability to customize and further optimize the analysis.

The ExplantAnalyzer algorithm in MATLAB (version 2021b) offers an automated approach for analyzing spiral ganglion explants [19], akin to Sholl analysis, by creating binary neurite images and summarizing primary neurite lengths. Tested with 50 ng/ ml BDNF-treated explants, its neurite length accuracy slightly trailed that of Sholl analysis (average deviation: 0 ± 28% vs. 1 ± 14%; supplemental raw data). Despite MATLAB’s power for algorithmic development and analysis, its complexity contrasts with Fiji’s user-friendliness. Both methods were similar in analysis time.

The study finds Sholl analysis with rm ANOVA to be the best automated technique for neurite growth assessment in spiral ganglion explants, outperforming manual methods in time and precision, and ideal for high-throughput demands. Additionally, Gray Value analysis adds value by assessing inner explant brightness, complementing insights from the Sholl analysis and making it perfect for extensive studies, enhancing efficiency and reducing manual effort. Finally, the evaluation of a 31-measurement point interval in the Sholl analysis with rm ANOVA represents a new and effective evaluation method, which can also be applied to other neuronal explants.

## Supporting information

S1 Fig Measurement results from the manual neurite tracing with NeuronJ and manual neuron count. A) The neurite number of primaries differ between the test groups. A significant difference between negative control and 10 ng/ ml BDNF (p = 0.045), 25 ng/ ml BDNF (p = 0.01) and 50 ng/ml BDNF (p < 0.001) was observed, F(3,8) = 11.486 p = 0.003. The number of branches distinguished the treatment groups between negative control and 10 ng/ ml BDNF (p = 0.004), 25 ng/ ml and 50 ng/ml BDNF (p < 0.001), F(3,8) = 10.290 p = 0.004. B) Diagram of mean length of primary neurites and branches. The mean length of primaries differed between negative control and 10 ng/ ml BDNF (p = 0.042), F(3,8) = 2.718 p = 0,115. The mean length of branches was not significant to each other, F(3,8) = 0.953 p = 0,46. C) neuronal survival within the explant. Manual count of neurons. In the 25 ng/ ml BDNF (p = 0.014) and 50 ng/ml BDNF (p = 0.037) group more neurons survived, than in the negative control, F(3,8) = 8.791 p = 0.007.
(TIF)

S2 Fig Additional information for Gray Value analysis (A, B), Sholl analysis (C-E). A) Diagram of brightness per ring of the interval ≤ 400 µm, source data for the normalization. The threshold distance, that represented the distance at which the brightness falls below the value of one, was drawn in a gray line. B) The normalized brightness of the explants is shown (Gray Value analysis). This brightness included neuronal cell bodies and neurites inside the explant. The difference between the negative control and 25 ng/ ml BDNF was significant (p = 0.024), ANOVA F(3,8) = 5.82 p = 0.021. C) Diagram of intersections per radius of the interval ≤ 400 µm, source data for the normalization. D) The normalized mean distance of the intersections from the explant is shown (Sholl analysis). The difference between negative control and 10 ng/ ml BDNF (p = 0.035) and 50 ng/ml BDNF (p = 0.036) was significant, ANOVA F(3,8) = 3.970 p = 0.053. E) Neurite length index was calculated from measurements of the Sholl analysis, ANOVA F(3,8) =5.74 p = 0.023. The difference between negative control and all BDNF concentrations (10 ng/ ml BDNF p = 0.015, 25 ng/ ml BDNF p = 0.017, 50 ng/ml BDNF p < 0.001) was significant. The neurite length index of high BDNF concentration was longer than that of lower BDNF concentrations (10 ng/ ml BDNF p = 0.019, 25 ng/ ml BDNF p = 0.021).
(TIF)

S3 Fig Illustration of covariates: experimental run (A), explant size (B). The data of the 50 ng/ml BDNF group were used (A, B). The gray value (brightness) of primary neurites and branchings was measured by NeuronJ plugin. A) Brightness of neurites for each experimental run, data of the 50 ng/ml BDNF group were compared. The neurites of the third experimental run were significantly brighter than those of the first two runs (ANOVA F(2,19) primaries F = 24.009 p < 0.001 and branching F = 21.379 p < 0.001). B) Dot plot of the marginal number of intersections and the explant size of the 50 ng/ml BDNF group. Larger explants had a higher number of intersections per radius (Pearson r = 0.603, p = 0.017).
(TIF)

S1 Table Measurement results of the manual evaluation (NeuronJ), the Gray Value and Sholl analyses. The following parameters were presented for the measurement methods: sum of primary neurite lengths, sum of branching lengths, neuronal survival, brightness per ring, threshold distance, inner explant brightness, intersections per radius, sum of intersections, neurite length index. The Sholl analysis (intersections within an interval 400 µm, sum of intersections) was as sensitive as the manual measurement (sum length of primary or branching). The significant difference between the Treatment groups was visualized. Results were shown in the corresponding measurement units or percentages normalized to the 50 ng/ ml BDNF group. The brightness of the 8-bit pictures was indicated as a value between 0 and 255. Mean values with SD were shown. For intersections and brightness, the marginal means were shown with SD. The multiple comparisons of two groups were shown in brackets. Significance levels were marked with asterisks: p < 0.001 (***), p < 0.01 (**), p < 0.5 (*).
(TIF)

S2 Table Comparison of the manual analysis method with the automated Gray Value and Sholl analysis. In the present study, the most suitable parameters for neurite outgrowth were compared. Analysis time was measured for raw data generation; the Sholl analysis was significantly faster than the manual method, had higher precision, provided high accuracy of results and a significant difference between treatment groups. Compared to the other Sholl analysis parameters, intersections per radius had advantages in the accuracy. The distinction of treatment groups was better for the intersections per radius than for the neurite length index.
(TIF)

S1 File Supplemental protocol for Sholl and Gray Value analysis using ImageJ Fiji. This document provides a step-by-step guide for performing Sholl and Gray Value analyses on neurite outgrowth images, including image preparation, processing, and data extraction.
(PDF)

S1 Raw data Raw experimental data collected from the neurite outgrowth of spiral ganglion explants. The dataset includes measurements from manual assessments, Sholl and Gray Value analyses, evaluations using the Explant Analyser, and statistical data for accuracy and precision.
(XLSX)
