# Supplementary figures and images for: Technical report: Efficient and accurate assessment of neurite outgrowth in spiral ganglion explants using Sholl analysis and repeated measurement ANOVA
Source: PLoS One. 2025 Jun 4;20(6):e0318613. doi: 10.1371/journal.pone.0318613 (PMC12136451; doi:10.1371/journal.pone.0318613)

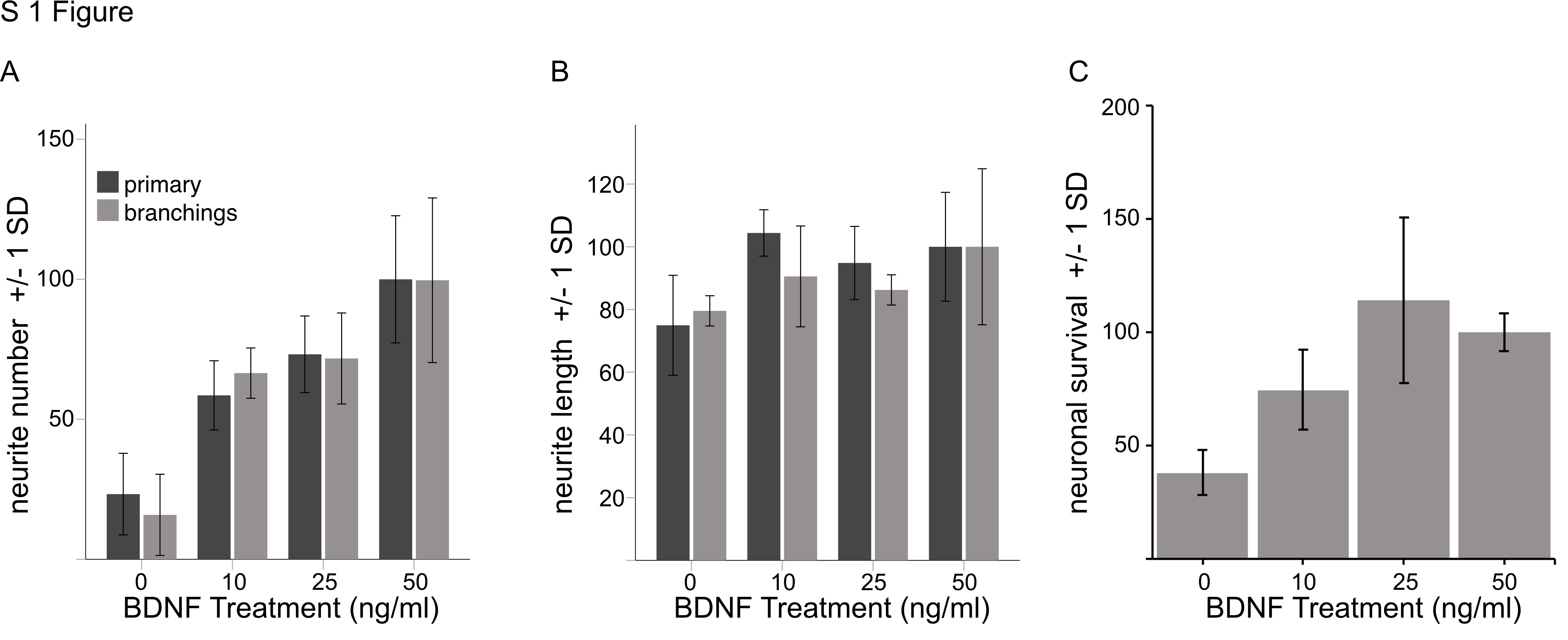

Supplement: S1 Fig — A) The neurite number of primaries differ between the test groups. A significant difference between negative control and 10 ng/ ml BDNF (p = 0.045), 25 ng/ ml BDNF (p = 0.01) and 50 ng/ml BDNF (p < 0.001) was observed, F(3,8) = 11.486 p = 0.003. The number of branches distinguished the treatment groups between negative control and 10 ng/ ml BDNF (p = 0.004), 25 ng/ ml and 50 ng/ml BDNF (p < 0.001), F(3,8) = 10.290 p = 0.004. B) Diagram of mean length of primary neurites and branches. The mean length of primaries differed between negative control and 10 ng/ ml BDNF (p = 0.042), F(3,8) = 2.718 p = 0,115. The mean length of branches was not significant to each other, F(3,8) = 0.953 p = 0,46. C) neuronal survival within the explant. Manual count of neurons. In the 25 ng/ ml BDNF (p = 0.014) and 50 ng/ml BDNF (p = 0.037) group more neurons survived, than in the negative control, F(3,8) = 8.791 p = 0.007. (TIF) [file pone.0318613.s001.tif]

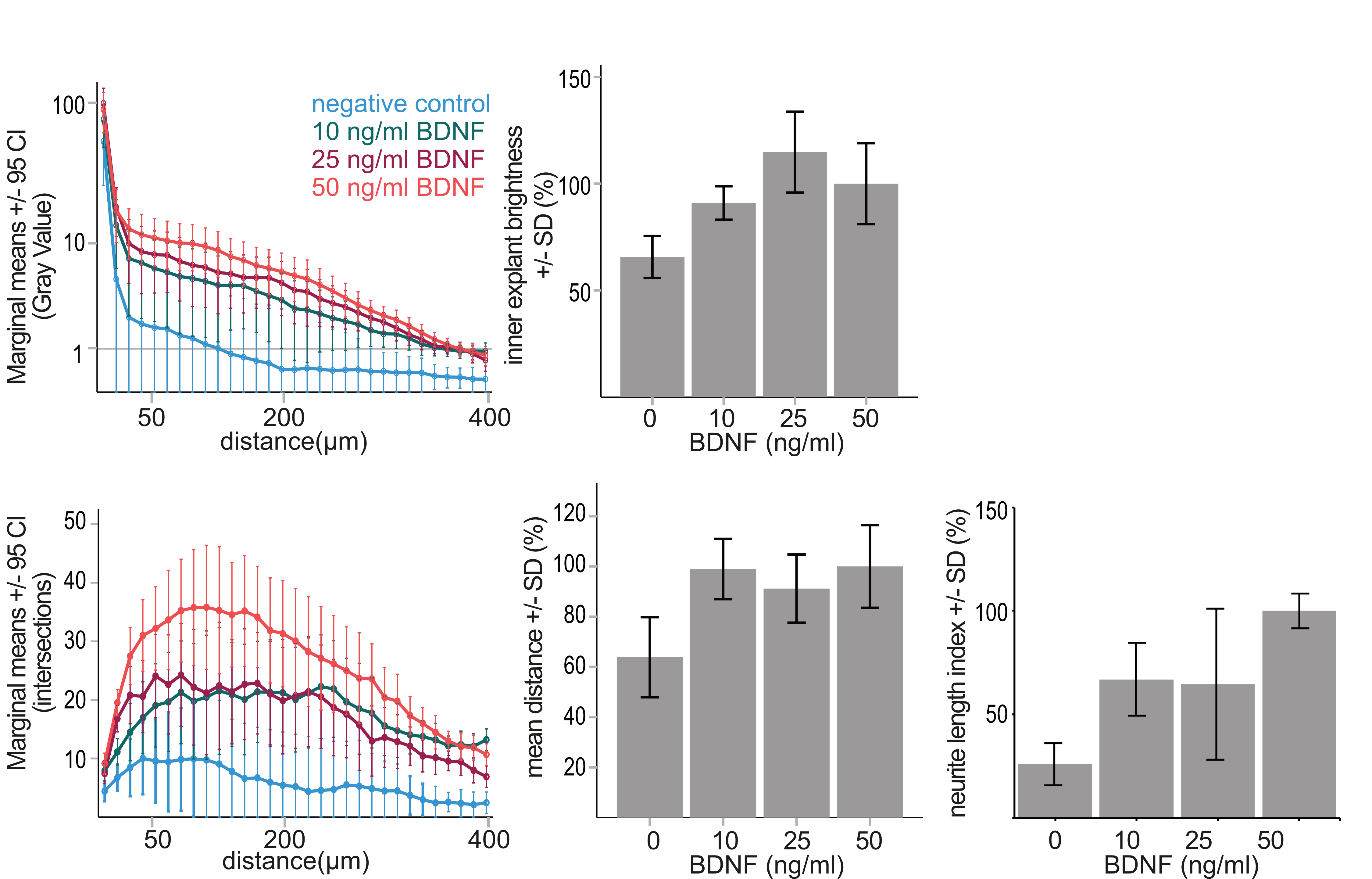

Supplement: S2 Fig — A) Diagram of brightness per ring of the interval ≤ 400 µm, source data for the normalization. The threshold distance, that represented the distance at which the brightness falls below the value of one, was drawn in a gray line. B) The normalized brightness of the explants is shown (Gray Value analysis). This brightness included neuronal cell bodies and neurites inside the explant. The difference between the negative control and 25 ng/ ml BDNF was significant (p = 0.024), ANOVA F(3,8) = 5.82 p = 0.021. C) Diagram of intersections per radius of the interval ≤ 400 µm, source data for the normalization. D) The normalized mean distance of the intersections from the explant is shown (Sholl analysis). The difference between negative control and 10 ng/ ml BDNF (p = 0.035) and 50 ng/ml BDNF (p = 0.036) was significant, ANOVA F(3,8) = 3.970 p = 0.053. E) Neurite length index was calculated from measurements of the Sholl analysis, ANOVA F(3,8) =5.74 p = 0.023. The difference between negative control and all BDNF concentrations (10 ng/ ml BDNF p = 0.015, 25 ng/ ml BDNF p = 0.017, 50 ng/ml BDNF p < 0.001) was significant. The neurite length index of high BDNF concentration was longer than that of lower BDNF concentrations (10 ng/ ml BDNF p = 0.019, 25 ng/ ml BDNF p = 0.021). (TIF) [file pone.0318613.s002.tif]

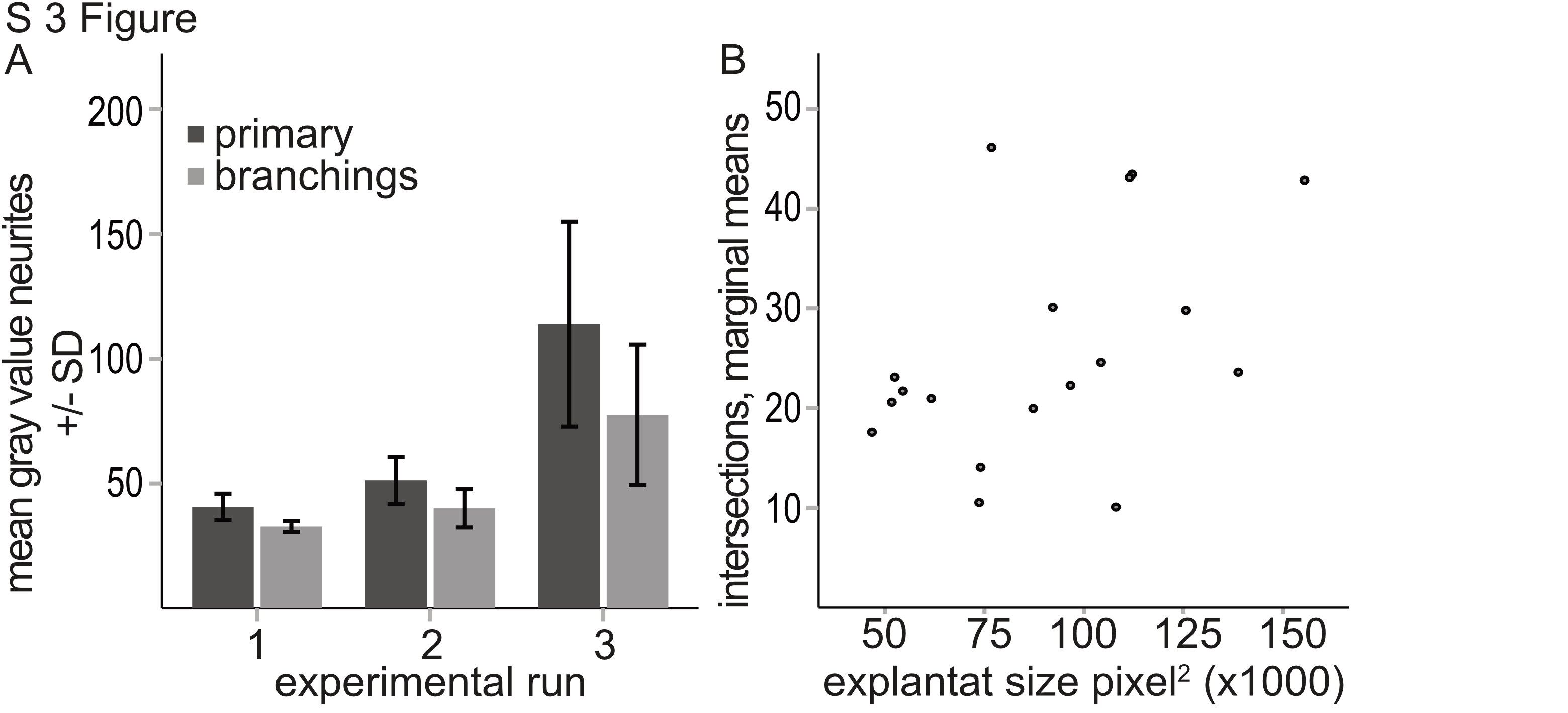

Supplement: S3 Fig — The data of the 50 ng/ml BDNF group were used (A, B). The gray value (brightness) of primary neurites and branchings was measured by NeuronJ plugin. A) Brightness of neurites for each experimental run, data of the 50 ng/ml BDNF group were compared. The neurites of the third experimental run were significantly brighter than those of the first two runs (ANOVA F(2,19) primaries F = 24.009 p < 0.001 and branching F = 21.379 p < 0.001). B) Dot plot of the marginal number of intersections and the explant size of the 50 ng/ml BDNF group. Larger explants had a higher number of intersections per radius (Pearson r = 0.603, p = 0.017). (TIF) [file pone.0318613.s003.tif]

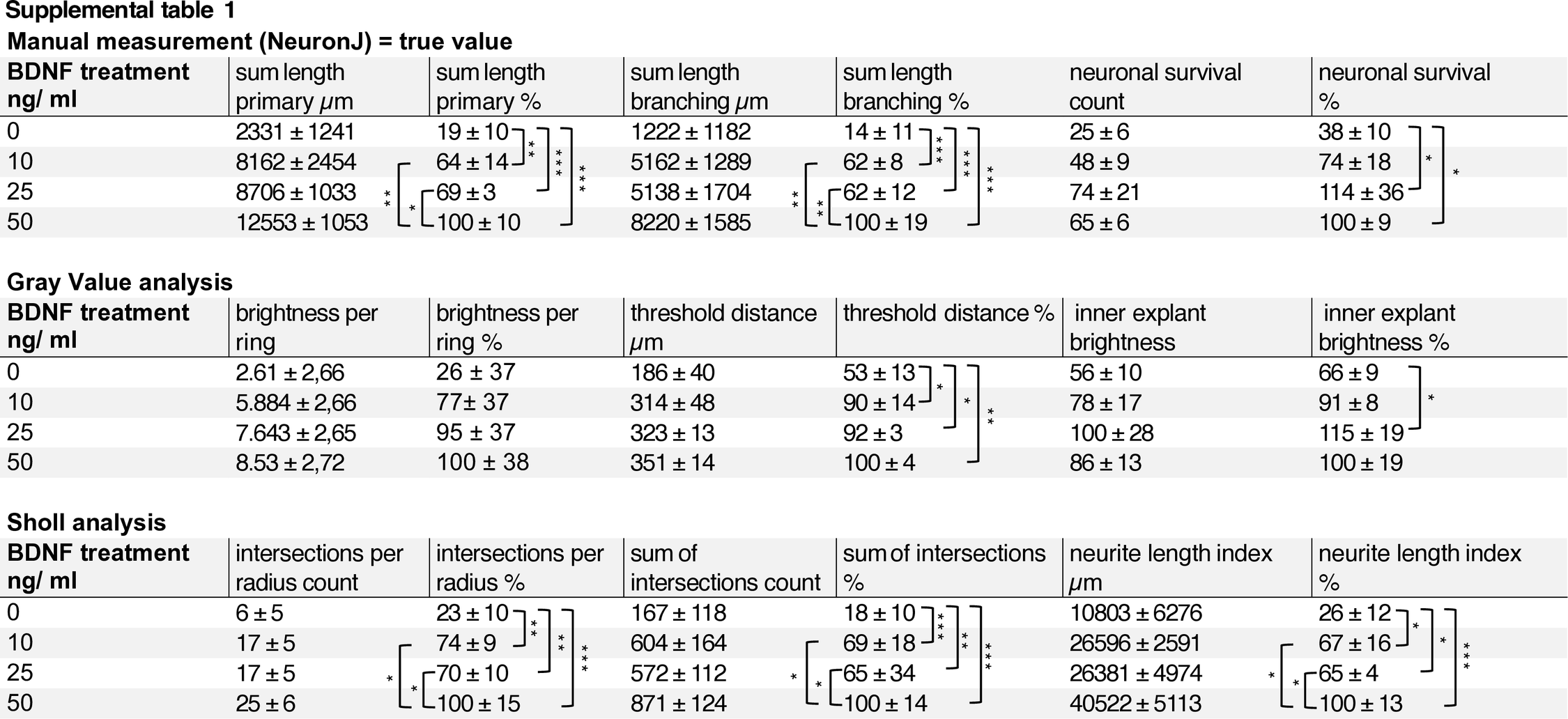

Supplement: S1 Table — The following parameters were presented for the measurement methods: sum of primary neurite lengths, sum of branching lengths, neuronal survival, brightness per ring, threshold distance, inner explant brightness, intersections per radius, sum of intersections, neurite length index. The Sholl analysis (intersections within an interval 400 µm, sum of intersections) was as sensitive as the manual measurement (sum length of primary or branching). The significant difference between the Treatment groups was visualized. Results were shown in the corresponding measurement units or percentages normalized to the 50 ng/ ml BDNF group. The brightness of the 8-bit pictures was indicated as a value between 0 and 255. Mean values with SD were shown. For intersections and brightness, the marginal means were shown with SD. The multiple comparisons of two groups were shown in brackets. Significance levels were marked with asterisks: p < 0.001 (***), p < 0.01 (**), p < 0.5 (*). (TIF) [file pone.0318613.s004.tif]

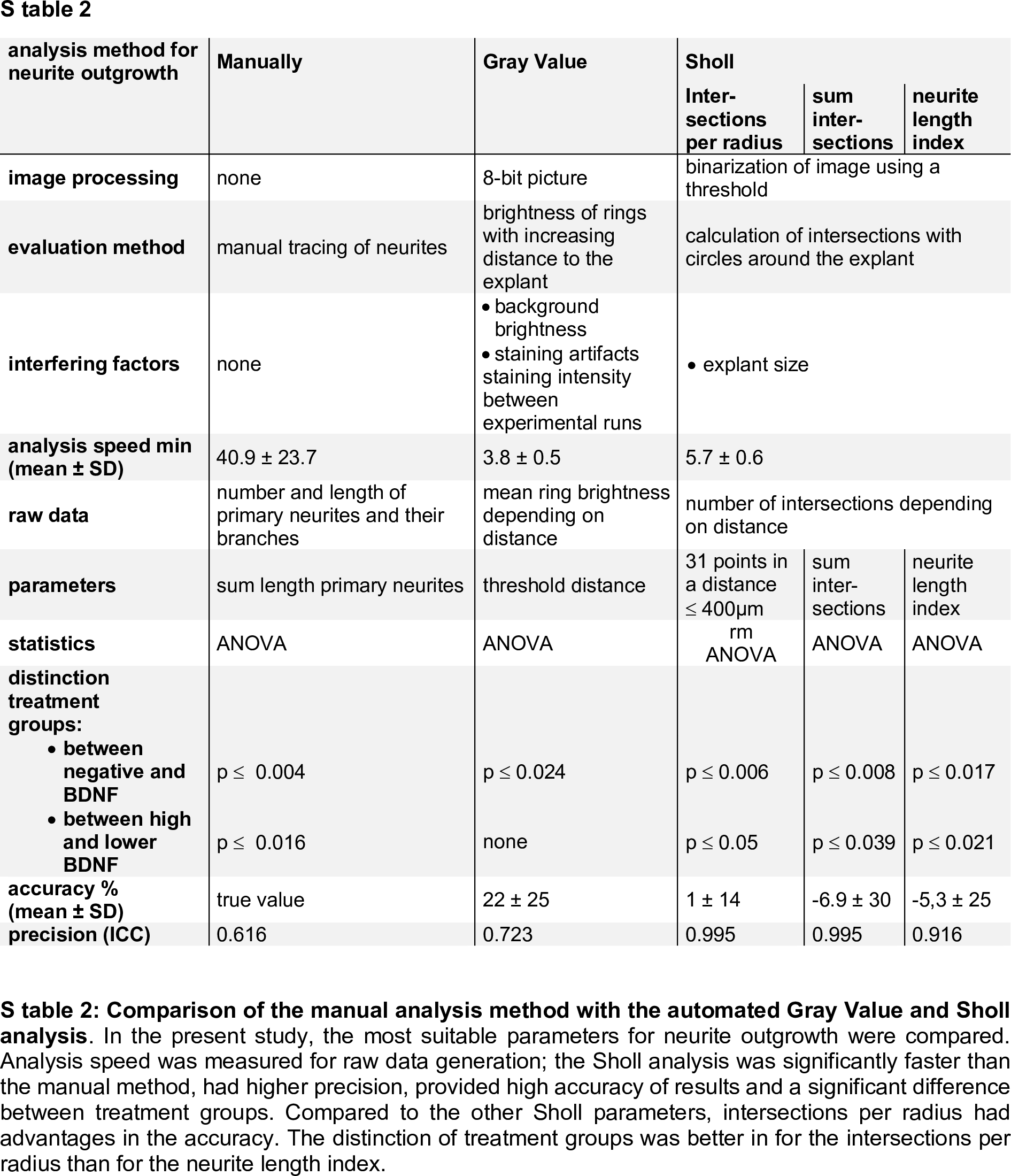

Supplement: S2 Table — In the present study, the most suitable parameters for neurite outgrowth were compared. Analysis time was measured for raw data generation; the Sholl analysis was significantly faster than the manual method, had higher precision, provided high accuracy of results and a significant difference between treatment groups. Compared to the other Sholl analysis parameters, intersections per radius had advantages in the accuracy. The distinction of treatment groups was better for the intersections per radius than for the neurite length index. (TIF) [file pone.0318613.s005.tif]
