# Supplementary material for: Technical report: Efficient and accurate assessment of neurite outgrowth in spiral ganglion explants using Sholl analysis and repeated measurement ANOVA
Source: PLoS One. 2025 Jun 4;20(6):e0318613. doi: 10.1371/journal.pone.0318613 (PMC12136451; doi:10.1371/journal.pone.0318613)
Supplement: S1 File — This document provides a step-by-step guide for performing Sholl and Gray Value analyses on neurite outgrowth images, including image preparation, processing, and data extraction. (PDF) [file pone.0318613.s006.pdf]

S1_file

Supplemental protocol for Sholl and Gray Value analysis using ImageJ Fiji (version 2.1.0)

1. Sholl analysis

1.1. Preliminary preparation for Sholl analysis

```
//open Tuj1 staining image (neurite outgrowth picture)
//set scale to pixel:
run("Set Scale...");
//measure background brightness using 4 rectangles at image corners and calculate mean:
setTool("rectangle");
run("Measure");
// increases picture size (circles must not touch image boarders):
run("Canvas Size...", "width=2500 height=2500 position=Center");

//open nuclear DAPI staining image (explant size)
//open Wand Tool and mark explant outlines
//adjust tolerance to outline whole explant

//copy cut-out with DAPI explant, paste to neurite outgrowth image, remove DAPI staining cut-out (only outlines remain):
run("Copy");
run("Paste");
run("Undo");
setTool("rectangle");
//place DAPI cut-out outlines onto the explant, enlarge outlines by 5 pixel to cover explant completely, measure size and cut to remove explant:
run("Enlarge...", "enlarge=5");
run("Measure");
run("Cut");
//noise reduction:
run("Despeckle");
//make a binary, personal decision on lower threshold:
run("Threshold...");
setThreshold(15, 255);
setOption("BlackBackground", false);
run("Convert to Mask");
// make a point in the centre of the explant using measurement data explant cut-out:
setTool("point");
makePoint(1275, 1230, "small yellow hybrid");
```

1.2. Sholl analysis implementation

```
// run plugins - neuroanatomy - Sholl (analysis from image), define start radius, step size (10 pixel), end radius using preview):
run("Sholl Analysis (From Image)...", "startradius=174.0 stepsize=10 endradius=484.0 hemishellchoice=[None. Use full shells] previewshells=false nspans=1.0 nspansintchoice=N/A primarybrancheschoice=[Infer from starting
```

```
radius] primarybranches=0.0 polynomialchoice=['Best fitting' degree]
polynomialdegree=0.0 normalizationmethoddescription=[Automatically
choose] normalizerdescription=Default plotoutputdescription=[Linear plot]
tableoutputdescription=[Detailed table] annotationsdescription=[ROIs (points
and 2D shells)] lutchoice=mpl-viridis.lut
luttable=net.imglib2.display.ColorTable8@62cd49d1 save=false
analysisaction=[Analyze image]");
// If necessary, remove intersections with staining artifacts using paintbrush
tool in background color and rerun Sholl analysis
setForegroundColor(255, 255, 255);
//copy first data (distance, intersections) to Microsoft Excel
```

1. Gray Value analysis

1.1. Preliminary preparation for Gray Value analysis

```
//open Tuj1 staining image (neurite outgrowth picture)
//set scale to pixel:
run("Set Scale...");
//measure background brightness using 4 rectangles at image corners and
calculate mean:
setTool("rectangle");
run("Measure");
//open nuclear DAPI staining image (explant size)
//open Wand Tool and mark explant
//adjust tolerance to outline whole explant
//copy cut-out with DAPI explant, paste to neurite outgrowth image,
remove DAPI staining cut-out (only outlines remain):
run("Copy");
run("Paste");
run("Undo");
setTool("rectangle");
```

1.2. Gray Value analysis implementation

```
//place DAPI cut-out outlines onto the explant, enlarge outlines to cover
explant completely, measure brightness (= explant brightness)
run("Enlarge...", "enlarge=5");
run("Measure");
// enlarge outlines and measure brightness (repeat this process until all
neurites are completely covered):
run("Enlarge...", "enlarge=10");
run("Measure");
run("Enlarge...", "enlarge=10");
run("Measure");
run("Enlarge...", "enlarge=10");
run("Measure");
…
//copy data (area, cumulative brightness) to Microsoft Excel
//calculate ring area by subtracting the previous area
//calculation of the cumulative ring brightness: subtracting the cumulative
brightness of the previous area
//calculation of the mean ring brightness: dividing the cumulative ring
brightness by the ring area
```
